# Serum levels of proinflammatory cytokines and selected bioelements in perimenopausal women with regard to body mass index

**DOI:** 10.18632/aging.203754

**Published:** 2021-12-10

**Authors:** Anna Maria Cybulska, Kamila Rachubińska, Małgorzata Szkup, Daria Schneider-Matyka, Irena Baranowska-Bosiacka, Dariusz Chlubek, Anna Lubkowska, Mariusz Panczyk, Joanna Sołek-Pastuszka, Elżbieta Grochans

**Affiliations:** 1 Department of Nursing, Pomeranian Medical University, Szczecin, Poland; 2 Department of Biochemistry and Medical Chemistry, Pomeranian Medical University, Szczecin, Poland; 3 Department of Functional Diagnostics and Physical Medicine, Pomeranian Medical University, Szczecin, Poland; 4 Division of Teaching and Outcomes of Education, Faculty of Health Science, Medical University of Warsaw, Warsaw, Poland; 5 Department of Anaesthesiology and Intensive Therapy, Pomeranian Medical University, Szczecin, Poland

**Keywords:** proinflammatory cytokines, bioelements, perimenopausal women, body mass index, menopause

## Abstract

During the menopause, decreased estrogen levels may be accompanied by increased levels of inflammatory mediators. Many studies also show significant relationships between the levels of bioelements and proinflammatory cytokines. The aim of this study was to assess the levels of proinflammatory cytokines, C-reactive protein (CRP), and selected bioelements in perimenopausal women with regard to BMI. Methods: The study of 217 perimenopausal women involved the completion of questionnaires concerning sociodemographic and medical data, anthropometric measurements, and blood collection. Results: In all studied women, the levels of IL-1β significantly positively correlated with Ca, Mg, and Sr; IFNγ significantly negatively correlated with Sr, and IL-6 with Mg. In women with a normal BMI, the levels of IL-1β significantly positively correlated with Ca and Sr, and CRP positively correlated with Zn. In overweight women, the levels of IL-1β positively correlated with Ca, IL-6 with Na, and IFNγ negatively correlated with Sr. In obese women, the levels of CRP positively correlated with Zn, TNFα with Mg, IFNγ with Cu and P. The levels of IL-6 negatively correlated with Ca and Mg. Conclusions: BMI may be one of the factors that affect the relationship between serum bioelement levels and the levels of proinflammatory cytokines and CRP in women, especially during the menopausal period.

## INTRODUCTION

Proinflammatory cytokines such as interleukin 1α (IL-1α), interleukin 1β (IL-1β), interleukin 6 (IL-6), tumor necrosis factor alpha (TNFα) and interferon γ (IFNγ) may play a role in the development of metabolic disorders, significantly affecting energy balance, as well as lipid and carbohydrate metabolism [1, 2].

Cytokines belonging to the interleukin 1 (IL-1) family play an important role in regulating the immune response [3]. They are described as endogenous pyrogenic factors, and mediators of leukocytes, lymphocytes, pancreatic β cells, and osteoclasts. They also take part in the regulation of cell growth, tissue regeneration and the development of chronic inflammation [4]. However, it is believed that the main role of the IL-1 family cytokines (including IL-1α and -1β) is to participate in inflammatory processes, in enhancing pain perception, and in the pathogenesis of many diseases. IL-1α is associated with the cell membrane and hence exhibits local effects, whereas IL-1β is secreted into the blood, acting systemically and is specifically associated with proinflammatory effects. IL-1β also affects the specific response processes, inducing the secretion of IL-6 by T lymphocytes, and influencing the development of B lymphocytes. IL-1β activates leukocytes and many other cells that are not directly related to the immune system, as it is also involved in tissue remodeling and acute phase protein synthesis [5].

IL-6 is one of the main proinflammatory cytokines responsible for the activation of the immune response, which may affect the hormonal balance and may also be responsible for some endocrine disorders and an increase in the level of free fatty acids in the blood. Therefore, elevated levels of this interleukin are observed in patients with dyslipidemia or insulin resistance. Many studies have confirmed a positive correlation between the value of the body mass index (BMI) and the level of IL-6 [6].

TNFα, on the other hand, significantly affects the metabolism and function of adipose tissue, and inhibits lipoprotein lipase (LPL) both at the level of mRNA and protein expression. It also affects the expression of two important regulators of adipose tissue differentiation: the CCAAT transcription factor and the nuclear receptor—peroxisome proliferator-activated receptor gamma 2 (PPARγ-2). The consequences of this include decreased expression of adipose tissue proteins—LPL, adipocyte lipoprotein-binding protein (AP2), and fatty acid synthase [7].

The level of C-reactive protein (CRP) in the human body increases with inflammation, which is a consequence of tissue damage. Thus, CRP is part of the positive acute phase proteins. C-reactive protein plays an important role in the immune system as one of its components. Its main task is to recognize microorganisms and detect cellular damage [8].

During the menopause, along with decreasing estrogen levels, higher levels of circulating interleukins (including IL-6), TNFα [3, 4, 9] and CRP are observed. The mechanism underlying the elevated markers of inflammation in postmenopausal women is unclear but may involve the production of inflammatory markers by adipose tissue or the effect of inflammatory markers on insulin-stimulated glucose utilization [10]. Furthermore, during the menopause, women experience deleterious changes in circulating markers of inflammation that are correlated with increased visceral obesity [11]. Estradiol and progesterone regulate TNFα, so a decline in hormone levels during the menopause may affect the production of inflammatory markers [10, 12]. However, there is no conclusive information on menopause-related differences in inflammatory markers. Nonetheless, there is growing evidence that chronic inflammation is a key element in the development of atherosclerotic cardiovascular disease, which is closely related to obesity, insulin resistance, and metabolic syndrome [13–16].

Many studies indicate a significant relationship between the levels of proinflammatory cytokines and the levels of some elements in the blood [9]. Elevated levels of elements in the blood often have various consequences, but their association with metabolic changes in obesity is not well documented. These relationships may be of clinical relevance, as excessive food consumption, food quality and environmental pollution are factors linked to obesity, hyperglycemia, and diabetes [17]. Essential trace elements play an important role in the proper functioning of metabolic enzymes. Increased intake and/or storage of certain elements may predispose to comorbidities associated with obesity and metabolic changes [18]. Overweight and obesity were also found to be significantly associated with iron deficiency [19]. Cytokines, on the other hand, secreted by various cells in the immune response, regulate a wide range of biological processes associated with innate and adaptive immunity. In the absence of infection or tissue injury, cytokines have been shown to play a significant role in the regulation of food intake and energy homeostasis. In the metabolic context, in obesity, local and systemic production of proinflammatory cytokines is altered and elevated levels of IL-1β, IL-6 and TNF-α impair systemic insulin sensitivity. Mild inflammation contributes to dysregulation of the metabolism of glucose and lipids associated with obesity [20].

The aim of this study was to assess the levels of proinflammatory cytokines (IL-1α, IL-1β, IL-6, TNFα and IFNγ), C-reactive protein (CRP), and selected elements (Ca, P, Na, K, Fe, Mg, Cu, Zn, Sr) in perimenopausal women with regard to BMI.

## RESULTS

### Characteristics of the study sample

A total of 217 women at the mean age of 52 (SD = ± 5), median age of 52, minimum age of 41 and maximum age of 66 participated in the study. The vast majority of the respondents had higher education (57.1%), lived in cities with a population of over 100,000 people (74.1%), were in a formal relationship (69.1%), and were employed (89.4%). Moreover, 56.7% were still menstruating. The analysis revealed that the mean body weight of the studied women was 72.65 (SD = 14.2) kg, the median weight was 71 kg, the minimum weight was 42.5 kg, and the maximum weight was 160 kg. The mean BMI of the respondents was 26.7 (SD = 4.8), the median was 26, the minimum value was 17.5, and the maximum value was 51.7. Analysis of BMI according to WHO guidelines showed that the vast majority of the respondents were normal weight (40.1%) or overweight (40.1%), while 19.8% were obese.

The blood levels of selected bioelements, proinflammatory cytokines, and CRP were determined. The analysis of the collected data demonstrated that the mean levels of bioelements in the blood of the studied women were within the normal ranges, however in some cases the levels of bioelements were below or above normal (Tables 1, 2).

**Table 1:** Serum levels of interleukins and CRP in all studied women.

**Inflammation parameters**	**M**	**SD**	**Mdn**	**IQR/2**	**Min-Max**
IL-1α [pg/ml]	8.09	38.06	2.42	0.32	1.50–431
IL-β [pg/ml]	243.34	305.25	79.40	265.08	0.34–998.3
IL-6 [pg/ml]	38.02	92.15	13.09	10.08	1.99–594.2
TNFα [pg/ml]	7.05	10.14	4.63	2.52	0.69–81.10
IFNγ [IU/ml]	0.11	0.34	0.03	0.01	0.03–3.85
CRP [mg/l]	2.52	4.88	1.27	0.68	0.99–65.06

Abbreviations: M: mean; SD: standard deviation; Mdn: median; IQR/2: quartile deviation.

**Table 2:** Serum levels of bioelements in all studied women.

**Bioelement levels**	**M**	**SD**	**Mdn**	**IQR/2**	**Min-Max**
Ca [mg/l]	94.06	27.23	88.73	19.00	36.35–192.59
P [mg/l]	107.16	34.02	103.79	18.52	47.63–248.61
Na [mg/l]	2200.09	1206.32	2061.86	350.02	839.98–13254.07
K [mg/l]	253.02	189.49	195.20	78.40	62.02–1263.13
Fe [mg/l]	1.28	0.55	1.22	0.25	0.47–4.87
Mg [mg/l]	15.75	4.10	15.42	2.50	7.25–30.51
Cu [mg/l]	0.82	0.29	0.77	0.16	0.26–2.15
Zn [mg/l]	2.01	0.94	1.84	0.51	0.63–6.18
Sr [mg/l]	0.15	0.08	0.11	0.07	0.00–0.37

Abbreviations: M: mean; SD: standard deviation; Mdn: median; IQR/2: quartile deviation.

The levels of selected bioelements, proinflammatory cytokines, and CRP were analyzed with regard to BMI (normal, overweight, obese). Statistically significant differences were found between BMI classifications according to WHO and the levels of IFNγ and CRP (Table 3). We observed that overweight women had significantly higher IFNγ levels than other women, and CRP levels were positively correlated with BMI values.

**Table 3:** Serum levels of interleukins and CRP with regard to BMI (normal, overweight, obese).

**Inflammation parameters**	**Normal weight**		**Overweight**		**Obesity**		**F**	*p* ^*^
	**M**	**SD**	**M**	**SD**	**M**	**SD**		
IL-1α [pg/ml]	10.99	52.02	7.72	30.11	2.99	3.01	0.641	0.528
IL-β [pg/ml]	273.07	318.27	224.24	306.37	221.84	276.65	0.688	0.504
IL-6 [pg/ml]	32.57	79.82	44.16	104.02	36.62	91.26	0.348	0.707
TNFα [pg/ml]	6.24	8.27	7.00	10.90	8.81	11.85	0.930	0.396
IFNγ [IU/ml]	0.06	0.09	0.18	0.51	0.05	0.05	3.885	**0.022**
CRP [mg/l]	1.65	1.94	2.74	7.07	3.88	3.20	3.105	**0.047**

Abbreviations: F: Fisher’s exact test; *p*: statistical significance ^*^ANOVA.

No statistically significant differences were observed between the levels of selected bioelements and BMI classifications according to WHO (normal, overweight, obese) (Table 4).

**Table 4:** Serum levels of bioelements with regard to BMI (normal, overweight, obese).

**Bioelement levels**	**Normal weight**		**Overweight**		**Obesity**		**F**	*p* ^*^
	**M**	**SD**	**M**	**SD**	**M**	**SD**		
Ca [mg/l]	97.73	29.75	91.14	26.04	92.55	23.78	1.360	0.259
P [mg/l]	108.31	37.82	105.88	31.91	107.42	30.56	0.112	0.894
Na [mg/l]	2305.16	1283.00	2119.23	1300.81	2151.12	780.87	0.559	0.573
K [mg/l]	246.92	191.62	254.57	190.56	262.19	186.97	0.097	0.907
Fe [mg/l]	1.25	0.42	1.27	0.56	1.39	0.72	0.987	0.374
Mg [mg/l]	16.18	4.14	15.51	4.43	15.39	3.27	0.783	0.459
Cu [mg/l]	0.82	0.32	0.79	0.25	0.87	0.29	1.190	0.306
Zn [mg/l]	1.91	0.83	2.17	1.12	1.89	0.71	2.214	0.112
Sr [mg/l]	0.15	0.08	0.15	0.08	0.15	0.08	0.265	0.767

Abbreviations: F: Fisher’s exact test; *p*: statistical significance ^*^ANOVA.

### Correlation between the levels of bioelements and the levels of proinflammatory cytokines and CRP

We analyzed the relationship between the levels of bioelements (Ca, P, Na, K, Fe, Mg, Cu, Zn, Sr), proinflammatory cytokines (IL-1α, IL-1β, IL-6, TNFα, and IFNγ), and CRP. Based on the results, statistically significant correlations were observed between the selected bioelements and three proinflammatory cytokines (IL-β, IFNγ, IL-6). The study demonstrated that IL-1β was positively correlated with the serum levels of Ca (r = 0.261, *p* = 0.000), Mg (r = 0.163, *p* = 0.017), and Sr (r = 0.229, *p* = 0.001). Moreover, negative correlations were observed between the levels of IFNγ and Sr (r = −0.151, *p* = 0.028), and between the serum levels of IL-6 and Mg (r = −0.149, *p* = 0.030) (Table 5).

**Table 5:** Correlations between the levels of bioelements and the levels of proinflammatory cytokines and CRP in the serum of all studied women.

**Bioelement levels**	**IL-1α [pg/ml]**	**IL-1β [pg/ml]**	**IL-6 [pg/ml]**	**TNFα [pg/ml]**	**IFNγ [IU/ml]**	**CRP [mg/l]**
Ca [mg/l]	−0.11	0.26^*^	−0.04	−0.01	−0.13	0.03
P [mg/l]	−0.02	−0.08	−0.12	−0.03	0.00	0.00
Na [mg/l]	−0.07	0.08	0.13	0.02	−0.02	−0.01
K [mg/l]	0.00	−0.06	−0.10	−0.09	−0.03	−0.06
Fe [mg/l]	−0.10	0.02	−0.04	−0.05	0.00	−0.07
Mg [mg/l]	−0.11	0.16^*^	−0.15^*^	0.07	−0.04	−0.01
Cu [mg/l]	−0.10	0.01	−0.13	0.00	−0.04	0.06
Zn [mg/l]	0.02	−0.09	0.06	0.08	0.04	0.01
Sr [mg/l]	−0.07	0.23^*^	0.09	−0.06	−0.15^*^	0.07

^*^p < 0.05 (two-tailed *p* value for Pearson′s r correlation).

Multivariate regression analysis of the influence of the bioelement levels on the levels of proinflammatory cytokines and CRP was performed. A statistically significant positive correlation was observed between the levels of Ca and IL-1β, and between the levels of Na and IL-6 in the serum (Table 6).

**Table 6:** Regression analysis of the influence of the levels of bioelements on the levels of proinflammatory cytokines and CRP in all studied women.

**Bioelement levels**	**IL-1α [pg/ml]**			**IL-1β [pg/ml]**			**IL-6 [pg/ml]**			**TNFα [pg/ml]**			**IFNγ [IU/ml]**			**CRP [mg/l]**		
	**β**	**t**	***p***	**β**	**t**	***p***	**β**	**t**	***p***	**β**	**t**	***p***	**β**	**t**	***p***	**β**	**t**	***p***
Ca [mg/l]	−0.01	−0.08	0.94	0.435	2.74	0.007	−0.2	−1.24	0.22	0.071	0.434	0.664	−0.066	−0.398	0.691	−0.133	−0.77	0.444
P [mg/l]	0.1	0.92	0.36	−0.152	−1.46	0.146	−0.07	−0.64	0.52	−0.155	−1.441	0.151	−0.008	−0.069	0.945	−0.003	−0.03	0.977
Na [mg/l]	−0.02	−0.33	0.74	0.001	0.007	0.994	0.24	3.21	0.002	0.039	0.512	0.609	0.026	0.347	0.729	−0.024	−0.31	0.757
K [mg/l]	−0.02	−0.26	0.79	−0.018	−0.25	0.800	−0.03	−0.45	0.65	−0.122	−1.671	0.096	−0.074	−0.993	0.322	−0.042	−0.55	0.583
Fe [mg/l]	−0.07	−0.87	0.39	−0.053	−0.71	0.478	0.01	0.13	0.896	−0.081	−1.037	0.301	0.042	0.536	0.593	−0.098	−1.24	0.217
Mg [mg/l]	−0.06	−0.58	0.56	0.013	0.14	0.887	−0.16	−1.63	0.1	0.134	1.379	0.169	0.052	0.528	0.598	−0.018	−0.18	0.855
Cu [mg/l]	−0.13	−1.17	0.24	−0.006	−0.06	0.955	−0.03	−0.32	0.74	0.04	0.366	0.715	−0.074	−0.67	0.503	0.187	1.68	0.094
Zn [mg/l]	0.08	1.01	0.32	−0.131	−1.79	0.075	0.14	1.94	0.054	0.136	1.801	0.073	0.091	1.195	0.234	−0.019	−0.24	0.808
Sr [mg/l]	−0.035	−0.266	0.791	0.369	−0.9	−0.114	0.197	1.538	0.126	−0.246	−1.869	0.06	−0.175	−1.318	0.189	0.207	1.47	0.143

Abbreviations: β: standardized regression coefficient; t: t statistics value, *p*: statistical significance.

We analyzed the relationships between the levels of bioelements and the levels of proinflammatory cytokines and CRP, taking into account the BMI of the studied women. In women with a normal BMI, a statistically significant correlation was observed between the levels of Ca and IL-1β (r = 0.29, *p* = 0.007), and between the levels of Sr and IL-1β (r = 0.29, *p* = 0.007). Moreover, there was a statistically significant correlation between the levels of Zn and CRP (r = 0.22, *p* = 0.042). No statistically significant correlations were observed between the levels of the remaining tested bioelements and the levels of proinflammatory cytokines and CRP in women with normal BMI. In overweight women, we found statistically significant correlations between the levels of Ca and IL-β (r = 0.27, *p* = 0.012), and between the levels of Na and IL-6 (r = 0.40, *p* < 0.001). There was also a correlation between the levels of Sr and IFNγ (r = −0.22, *p* = 0.041). No statistically significant correlations were observed between the levels of the remaining tested bioelements and the levels of proinflammatory cytokines and CRP in women with overweight. In obese women, statistically significant correlations were noted between the levels of Zn and CRP (r = 0.36, *p* = 0.022), and between the levels of Mg and TNFα (r = 0.37, *p* = 0.018). Statistically significant correlations were also observed between the levels of Cu and IFNγ (r = 0.45, *p* = 0.003), and between P and IFNγ (r = 0.32, *p* = 0.040). There were also statistically significant correlations between the levels of Ca and IL-6 (r = −0.32, *p* = 0.043), and between the levels of Mg and IL-6 (r = −0.52 *p* < 0.001). No statistically significant correlations were observed between the levels of the remaining tested bioelements and the levels of proinflammatory cytokines and CRP in obese women (Table 7).

**Table 7:** Analysis of the correlations between the levels of proinflammatory cytokines and the levels of bioelements in the serum of the studied women with regard to BMI.

**Bioelement levels**	**BMI–normal weight (*n* = 87)**						**BMI–overweight (*n* = 87)**						**BMI–obesity (*n* = 43)**					
	**IL-1α [pg/ ml]**	**IL-β [pg/ ml]**	**IL-6 [pg/ ml]**	**TNFα [pg/ ml]**	**IFNγ [IU/ ml]**	**CRP [mg/ l]**	**IL-1α [pg/ ml]**	**IL-β [pg/ ml]**	**IL-6 [pg/ ml]**	**TNFα [pg/ ml]**	**IFN [IU/ ml]**	**CRP [mg/ l]**	**IL-1α [pg/ ml]**	**IL-β [pg/ ml]**	**IL-6 [pg/ ml]**	**TNFα [pg/ ml]**	**IFNγ [IU/ ml]**	**CRP [mg/ l]**
Ca [mg/l]	−0.15	0.29^*^	−0.04	0.02	−0.17	0.01	−0.09	0.27^*^	0.07	−0.01	−0.16	0.11	−0.01	0.12	−0.32	−0.03	0.20	−0.21
P [mg/l]	−0.06	−0.08	−0.05	−0.02	−0.12	0.07	0.04	−0.09	−0.14	−0.03	0.03	−0.06	−0.06	−0.11	−0.24	−0.05	0.32	0.15
Na [mg/l]	−0.09	0.13	−0.06	−0.04	−0.06	0.02	−0.08	0.01	0.40^*^	0.04	−0.01	0.00	0.03	0.18	−0.29	0.16	0.24	−0.07
K [mg/l]	−0.03	−0.05	−0.09	−0.03	−0.13	−0.10	0.05	−0.07	−0.11	−0.10	−0.03	−0.05	0.00	−0.06	−0.12	−0.18	0.23	−0.16
Fe [mg/l]	−0.12	0.19	−0.01	−0.17	−0.14	0.09	−0.13	−0.06	0.01	0.03	0.01	−0.10	0.05	−0.08	−0.16	−0.10	0.21	−0.21
Mg [mg/l]	−0.13	0.13	−0.07	0.08	−0.19	0.07	−0.13	0.17	−0.09	−0.03	−0.03	0.03	0.07	0.21	−0.52	0.37	0.27	−0.30
Cu [mg/l]	−0.10	−0.02	−0.06	−0.05	−0.14	0.21	−0.11	0.06	−0.13	0.04	−0.03	0.01	−0.06	−0.05	−0.25	0.07	0.45	0.02
Zn [mg/l]	0.07	−0.09	−0.07	0.02	−0.12	0.22^*^	−0.03	−0.08	0.18	0.16	0.03	−0.08	−0.12	−0.15	−0.20	−0.03	0.03	0.36
Sr [mg/l]	−0.15	0.29^*^	0.06	−0.08	−0.10	−0.02	0.06	0.21	0.21	−0.04	−0.22^*^	0.18	−0.04	0.11	−0.13	−0.07	−0.12	−0.09

^*^p < 0.05, Pearson′s correlation.

We carried out multivariate regression analysis of the relationship between the levels of bioelements and the levels of proinflammatory cytokines and CRP with regard to the BMI of the studied women. The results showed that in the group of women with normal BMI the effect of Ca levels on TNFα was positive (β = 0.609, *p* = 0.045), while the effect of Fe (β = −0.363, *p* = 0.015) and Sr (β = −0.556, *p* = 0.021) levels was negative. We also observed a positive effect of Cu (β = 0.565, *p* = 0.004) and Zn (β = 0.282, *p* = 0.021) levels, and a negative effect of P levels (β = −0.455, *p* = 0.029) on the level of CRP (Table 8).

**Table 8:** Regression analysis of the influence of the levels of bioelements on the levels of proinflammatory cytokines and CRP in women with normal BMI.

**Bioelement levels**	**BMI–normal weight (*n* = 87)**																	
	**IL-1α [pg/ml]**			**IL-1β [pg/ml]**			**IL-6 [pg/ml]**			**TNFα [pg/ml]**			**IFNγ [IU/ml]**			**CRP [mg/l]**		
	**β**	**t**	***p***	**β**	**t**	***p***	**β**	**t**	***p***	**β**	**t**	***p***	**β**	**t**	***p***	**β**	**t**	***p***
Ca [mg/l]	0.236	0.760	0.450	0.252	0.851	0.398	−0.316	−1.002	0.319	**0.609**	**2.035**	**0.045**	0.017	0.055	0.956	−0.357	−1.219	0.227
P [mg/l]	0.035	0.163	0.871	−0.198	−0.958	0.341	0.109	0.496	0.621	0.018	0.085	0.933	0.113	0.520	0.605	**−0.455**	**−2.224**	**0.029**
Na [mg/l]	−0.009	−0.066	0.948	0.006	0.047	0.962	−0.036	−0.261	0.794	−0.057	−0.442	0.660	0.049	0.363	0.718	0.000	0.003	0.998
K [mg/l]	−0.084	−0.682	0.497	0.066	0.561	0.576	−0.059	−0.470	0.640	−0.114	−0.963	0.339	−0.154	−1.251	0.215	−0.102	−0.878	0.382
Fe [mg/l]	−0.072	−0.472	0.638	0.209	1.441	0.154	0.093	0.602	0.549	**−0.363**	**−2.478**	**0.015**	−0.051	−0.338	0.736	0.048	0.332	0.741
Mg [mg/l]	−0.111	−0.653	0.516	−0.069	−0.425	0.672	−0.007	−0.040	0.969	0.101	0.622	0.536	−0.125	−0.738	0.463	0.164	1.030	0.306
Cu [mg/l]	−0.173	−0.848	0.399	−0.018	−0.093	0.926	−0.017	−0.081	0.936	−0.113	−0.576	0.566	−0.135	−0.663	0.510	**0.565**	**2.943**	**0.004**
Zn [mg/l]	0.119	0.939	0.351	−0.084	−0.695	0.489	−0.078	−0.606	0.546	0.008	0.065	0.948	−0.085	−0.668	0.506	**0.282**	**2.356**	**0.021**
Sr [mg/l]	−0.331	−1.356	0.179	0.088	0.376	0.708	0.299	1.207	0.231	**−0.556**	**−2.362**	**0.021**	−0.111	−0.457	0.649	0.134	0.583	0.561

Abbreviations: β: standardized regression coefficient, t: t statistics value, *p*: statistical significance.

The study revealed that in the group of overweight women, Mg levels had a negative effect on the level of TNFα (β = −1.426, *p* = 0.007) (Table 9). In the case of obese women, a positive effect of Mg levels on TNFα levels (β = 1.985, *p* = 0.001) and a negative effect of Ca levels on TNFα levels (β = −1.839, *p* = 0.023) were observed. Based on the results, the effect of Na on IL-6 levels was positive in the group of overweight women (β = 0.677, *p* = 0.006). A negative effect of Mg on IL-6 levels was observed in obese subjects (β = −1.285, *p* = 0.022) (Table 10).

**Table 9:** Regression analysis of the influence of the levels of bioelements on the levels of proinflammatory cytokines and CRP in overweight women.

**Bioelement levels**	**BMI–overweight (*n* = 87)**																	
	**IL-1α [pg/ml]**			**IL-1β [pg/ml]**			**IL-6 [pg/ml]**			**TNFα [pg/ml]**			**IFNγ [IU/ml]**			**CRP [mg/l]**		
	**β**	**t**	***p***	**β**	**t**	***p***	**β**	**t**	***p***	**β**	**t**	***p***	**β**	**t**	***p***	**β**	**t**	***p***
Ca [mg/l]	−0.781	−0.946	0.345	0.916	1.163	0.246	0.102	0.134	0.894	0.493	0.629	0.530	−0.318	−0.394	0.694	0.556	0.678	0.498
P [mg/l]	0.367	0.717	0.474	−0.264	−0.541	0.589	−0.001	−0.003	0.998	−0.286	−0.588	0.557	−0.026	−0.052	0.959	−0.067	−0.135	0.892
Na [mg/l]	−0.074	−0.279	0.780	−0.241	−0.959	0.339	**0.677**	**2.788**	**0.006**	−0.113	−0.451	0.652	0.045	0.173	0.863	−0.115	−0.451	0.652
K [mg/l]	0.113	0.691	0.491	0.018	0.114	0.909	0.087	0.579	0.563	0.080	0.514	0.608	−0.181	−1.135	0.258	0.137	0,741	0.460
Fe [mg/l]	−0.049	−0.187	0.852	−0.443	−1.778	0.077	−0.193	−0.802	0.423	438.000	1.765	0.079	0.192	0.754	0.452	−0.230	−0.914	0.362
Mg [mg/l]	0.040	0.074	0.941	−0.264	−0.506	0.613	0.538	1.072	0.285	**−1.426**	**−2.750**	**0.007**	0.459	0.861	0.390	0.108	0.204	0.839
Cu [mg/l]	0.044	0.094	0.925	0.296	0.666	0.506	−0.309	−0.723	0.471	−0.007	−0.016	0.987	−0.523	−1.150	0.252	0.134	0.297	0.767
Zn [mg/l]	−0.174	−0.610	0.542	−0.115	−0.424	0.672	0.360	1.373	0.171	0.302	1.113	0.267	0.291	1.044	0.298	−0.547	−1.969	0.050
Sr [mg/l]	0.680	1.770	0.078	−0.158	−0.432	0.666	0.045	0.128	0.899	0.039	0.106	0.916	−0.624	−1.666	0.097	0.380	0.970	0.330

Abbreviations: β: standardized regression coefficient; t: t statistics value; *p*: statistical significance.

**Table 10:** Regression analysis of the influence of the levels of bioelements on the levels of proinflammatory cytokines and CRP in obese women.

**Bioelement levels**	**BMI–obesity (*n* = 43)**																	
	**IL-1α [pg/ml]**			**IL-1β [pg/ml]**			**IL-6 [pg/ml]**			**TNFα [pg/ml]**			**IFNγ [IU/ml]**			**CRP [mg/l]**		
	**β**	**t**	***p***	**β**	**t**	***p***	**β**	**t**	***p***	**β**	**t**	***p***	**β**	**t**	***p***	**β**	**t**	***p***
Ca [mg/l]	−0.012	−0.015	0.988	−0.526	−0.654	0.514	0.095	0.123	0.902	**−1.839**	**−2.297**	**0.023**	0.169	0.205	0.838	−0.746	−0.862	0.390
P [mg/l]	−0.195	−0.323	0.743	353.0	0.613	0.541	−0.546	−0.983	0.327	0.076	0.132	0.895	−0.007	−0.011	0.991	0.310	0.530	0.596
Na [mg/l]	0.033	0.095	0.924	0.318	0.928	0.355	−0.354	−1.071	0.285	0.344	1.008	0.315	−0.047	−0.134	0.893	0.175	0.505	0.614
K [mg/l]	0.026	0.158	0.874	−0.120	−0.754	0.452	−0.078	−0.512	0.609	−0.165	−1.047	0.297	0.113	0.696	0.487	−0.147	−0.682	0.496
Fe [mg/l]	0.126	0.512	0.603	−0.165	−0.701	0.484	0.076	0.333	0.739	−0.001	−0.006	0.995	−0.063	−0.262	0.793	0.011	0.045	0.964
Mg [mg/l]	0.204	0.336	0.738	0.607	1.048	0.296	**−1.285**	**−2.304**	**0.022**	**1.985**	**3.444**	**0.001**	−0.058	−0.097	0.923	−0.382	−0.639	0.523
Cu [mg/l]	0.210	0.442	0.659	−0.334	−0.738	0.461	0.384	0.881	0.379	0.288	0.639	0.523	0.334	0.721	0.472	−0.137	−0.299	0.765
Zn [mg/l]	−0.160	−0.476	0.635	−0.006	−0.018	0.985	−0.002	−0.007	0.994	−0.143	−0.447	0.655	−0.116	−0.352	0.725	0.567	1.726	0.086
Sr [mg/l]	0.047	0.130	0.897	−0.151	−0.439	0.661	−0.109	−0.329	0.743	0.240	0.703	0.483	0.251	0.714	0.476	0.005	0.012	0.990

Abbreviations: β: standardized regression coefficient; t: t statistics value; *p*: statistical significance.

## DISCUSSION

To our knowledge this is the first publication evaluating the influence of BMI on the levels of proinflammatory cytokines (IL-1α, IL-1β, IL-6, TNFα and IFNγ), CRP, and selected bioelements (Ca, P, Na, K, Fe, Mg, Cu, Zn, Sr) in the blood serum of perimenopausal women. In fact, no publication on a similar topic has been found in the literature to date.

Our study showed: a significant positive relationship between IL-1β and serum Ca, Mg, and Sr levels; a significant negative relationship between IFNγ and Sr levels; and a significant negative relationship between IL-6 and Mg levels in the serum of all studied women. Women with normal BMI showed a significant positive relationship between serum IL-1β levels and the levels of Ca and Sr. Moreover, a positive relationship was found between the levels of Zn and CRP. In overweight women, positive correlations were demonstrated between IL-1β levels and serum Ca levels, and between IL-6 levels and Na levels. A negative correlation was found between IFNγ and Sr levels. In obese women, a positive correlation was noted between serum CRP and Zn levels, TNFα and Mg levels, IFNγ and Cu and P levels. Moreover, in this group of women serum IL-6 levels negatively correlated with the levels of Ca and Mg.

A study by Luciano-Mateo F. et al. [18] showed a significant increase in serum levels of some trace elements (As, Ba, Cu, Se, and Sr), and decreased levels of Ca, Fe, Mg, Na, and Zn in women with morbid obesity. The authors observed a significant decline in Fe levels in women with morbid obesity. These data are consistent with the results of a meta-analysis confirming significant Fe deficiency in obese children and adults [19, 21]. Deficiencies may result from either low intake, or reduced Fe absorption or sequestration due to chronic inflammation [22–24].

Furthermore, Luciano-Mateo F. et al. [18] informed that patients diagnosed with type 2 diabetes had lower serum Mg levels than those in the control group. Moreover, Mg levels negatively correlated with blood glucose levels in obese women. The literature review shows that there are few data on changes in serum Mg levels with regard to BMI. One of the studies showing such a relationship is that of Oliveira et al. [25], who confirmed decreased serum Mg levels in obese individuals. In addition, the meta-analysis conducted by Wu et al. [26] revealed that low Mg levels were associated with a higher incidence of ischemic heart disease, hypertension, and type 2 diabetes.

According to Luciano-Mateo et al. serum Ca levels were reduced in obese women [18]. In a study by Pardhe et al. [27], serum Ca and estradiol levels in postmenopausal women were significantly lower, and serum *P* levels were significantly higher than in their premenopausal counterparts. The age of the subjects significantly correlated with bone Ca levels in postmenopausal women, while no such relationship was found in the premenopausal group. Similar results were obtained by LeKhi et al. [28], who noticed a significant decrease in serum Ca levels in postmenopausal women compared to premenopausal ones. An analogous trend for changes in biochemical markers of bone turnover was also reported in other studies [29–32].

In the study by Onyeukwu et al. [33], serum Ca levels were significantly lower in postmenopausal women compared to premenopausal respondents, but there was no significant difference in serum *P* levels between these groups. Szkup et al. [34] analyzed serum Mg, Zn, Ca, Cu, and Fe levels in postmenopausal women. They found that Mg levels were lowest in women with depressive symptoms (14.28 ± 2.13 mg/l), and highest in women without depressive symptoms (16.30 ± 3.51 mg/l). The mean serum Mg level (15.75 ± 3.23 mg/l) was below the reference values (18.77−24 mg/l). In the case of other bioelements, the mean serum Zn level was 0.70 ± 0.33 mg/l, Ca 72.59 ± 11.78 mg/l, Cu 1.10 ± 0.22 mg/l, and Fe 1.06 ± 0.36 mg/l. In our study, the serum levels of the tested bioelements in postmenopausal women were similar to the mean values as in the study quoted above.

A study by Luciano-Mateo F. et al. [18] also showed a strong association between Sr levels and severe obesity. However, it is worth pointing out that the search for the scientific literature revealed a significant lack of publications on this topic. We only noted reports that elevated Sr levels may have proinflammatory and atherosclerotic effects [35].

Many researchers claim that low Zn intake and low serum Zn levels significantly contribute to the growing prevalence of obesity and comorbidities [24, 36].

In our study, the mean levels of the tested elements, taking into account all studied menopausal women, did not diverge from the reference values. Both in the group with normal BMI, overweight and obesity, the mean levels of the studied elements were within normal ranges.

In general, cytokines constitute a large group of peptides and proteins that affect almost all biological processes in the body, are involved in signaling between immune cells, and play a key role in regulating fat metabolism. Cytokines are produced in response to inflammatory stimuli mainly by macrophages and Th cells, but also by other inflammatory cells, as well as vascular cells and adipocytes. Most adipokines are proinflammatory and promote the development of metabolic diseases. The main sources of IL-1α and IL-1β are indicator cells of the innate immune system (macrophages and monocytes) [37, 38]. IFNγ, on the other hand, is a cytokine produced primarily by immune cells, including ear-like lymphocyte (NK cells) populations, innate lymphoid cells (ILCs), and adaptive immune cells such as T helper 1 (TH1) cells and CD8 + cytotoxic lymphocytes (CTLs) [39].

Kim et al. [40] reported that the oldest group (52−63 years) of the studied menopausal women had the highest blood levels of IL-6, TNFα, Ilα, and IL-1β. In addition, significant interactions between age and menopause were observed for the serum levels of IL-6, TNFα, and IL-1β produced from peripheral blood mononuclear cells (PBMCs). Moreover, serum IL-6 levels were positively correlated with age, and the levels of TNFα and IL-1β. Ershler and Keller, on the other hand, observed that the increased production of cytokines in healthy older individuals may be due to the reduced levels of sex hormones [41]. Similar results were obtained by other researchers, who emphasized that estradiol inhibits proinflammatory cytokine gene expression, NF-κB binding and proinflammatory cytokine production [42, 43]. Also, a study by Malutan et al. [44] showed that serum levels of IL-1β, IL-8, and TNFα in naturally menopausal women and women with surgically induced menopause were significantly higher than in women of childbearing potential.

As stated by Smidt et al. [45], who evaluated the expression of Zn transport proteins from the Zipz and ZnTs families in visceral and subcutaneous adipose tissue of obese patients, most of these proteins are expressed at higher levels in subcutaneous adipose tissue. According to these authors, this high expression of Zn transport protein genes suggests that obesity may significantly affect the Zn transport system in adipose tissue. Also in our study, a significant positive relationship was found between CRP and Zn levels in the serum of obese women.

Moreover, many studies have confirmed that proinflammatory molecules secreted by adipose tissue such as IL-6, IL-8, and TNFα are present in high concentrations in obese individuals [45, 46]. In the study by Piva et al. [47], obese patients had elevated levels of IL-6. Different results were obtained by Feitosa et al. [48], who analyzed 20−50-year-old women. Both Park et al. [32] and Winkler et al. [49] observed that plasma TNFα levels in obese women were higher than in the control group.

In our investigation, the levels of the tested proinflammatory cytokines were similar to those in the cited studies. Moreover, in women with normal BMI, a positive effect of Ca levels on TNFα levels, and a negative effect of Fe and Sr levels on TNFα levels were noted. There was also a significant relationship between the level of CRP and the levels of Cu, Zn, and P.

Furthermore, we observed a significant association between the levels of proinflammatory cytokines and the levels of key elements in overweight women, in whom Il-6 levels were related to Na levels, which may contribute to the development of hypertension.

What is more, serum Ca and Mg levels negatively correlated with the level of IL-6 in a group of obese women, which may contribute to osteoporosis in the menopause, when estrogen levels are reduced.

Considering the complexity of mechanisms associated with changes in bioelement metabolism, especially in obese individuals, and the probable influence of proinflammatory cytokines on these changes, further studies should be conducted to evaluate the role of these molecules in the distribution of bioelements with regard to BMI.

The limitation of our study is a small size of the study sample. It is also worth noting that our results may diverge from the results reported by other authors due to the possible use of different methods for determining serum bioelement levels.

## CONCLUSIONS

BMI may be one of the factors that affect the relationship between serum bioelement levels and the levels of proinflammatory cytokines and CRP in women, especially during the menopausal period.

## MATERIALS AND METHODS

250 perimenopausal women living in the West Pomeranian Voivodeship were invited to take part in the study. The inclusion criteria were: age >45 years, consent to participate in the study, no history of menopausal hormone therapy (MHT), no history of current psychiatric treatment, no addictions (alcohol and cigarettes), use of a normal diet based on Polish cuisine without supplementation with micro- and macroelements. The exclusion criteria were: history of psychiatric problems, micro- and micronutrient supplementation, taking MHT, refusal to participate in the study, and incorrectly completed documentation.

Patients were recruited through leaflets placed in primary healthcare centers and specialist clinics in Szczecin. Finally, 217 women who met all the inclusion criteria were qualified for the study. Informed consent was obtained from all subjects involved in the study. The study was conducted according to the guidelines of the Declaration of Helsinki and approved by the Bioethics Committee of the Pomeranian Medical University in Szczecin [KB-0012/181/13].

### Procedure

#### Preliminary research

The research was conducted in 2019 in several stages. After obtaining the written consent, the patients completed a proprietary questionnaire, including questions about sociodemographic data (age, education, place of residence, marital status, employment) and medical data (last menstruation, use of dietary supplements, and MHT). The next step was to measure body weight [kg] and height [m] and calculate the body mass index (BMI). On the basis of BMI values, women were classified into the group with normal body weight (18.5−24.9), overweight (25.0−29.9), or obesity (BMI > 30.0) [50].

### Biochemical tests

#### Determination of serum Ca, P, Na, K, Fe, Mg, Cu, Zn, Sr levels

Samples were analyzed using inductively coupled plasma optical emission spectrometry (ICP-OES, ICAP 7400 Duo, Thermo Scientific) equipped with a concentric nebulizer and cyclonic spray chamber to determine their Ca, P, Na, K, Fe, Mg, Cu, Zn, Sr content. Analysis was performed in radial and axial mode. The samples were thawed in room temperature and digested using microwave digestion system MARS 5, CEM. The volume of the sample given to research was 0.75 ml. The samples were transferred to clean polypropylene tubes. 4 mL of 65% HNO_3_ (Suprapur, Merck) was added to each vial and each sample was allowed 30 minutes pre reaction time in the clean hood. After completion of the pre-reaction time, 1mL of non-stabilized 30% H_2_O_2_ solution (Suprapur, Merck) was added to each vial. Once the addition of all reagents was complete, the samples were placed in special Teflon vessels and heated in microwaved digestion system for 35 minutes in 180°C (15 min ramp to 180°C and maintained at 180°C for 20 min). At the end of digestion all samples were removed from the microwave and allowed to cool to room temperature. In the clean hood, samples were transferred to acid-washed 15 mL polypropylene sample tubes. A further 5-fold dilution was performed prior to ICP-OES measurement. The volume of 2 ml was taken from each digest. The samples were spiked with an internal standard to provide a final concentration of 0.5 mg/L Ytrium, 1 ml of 1% Triton (Triton X-100, Sigma) and diluted to the final volume of 10 mL with 0.075% nitric acid (Suprapur, Merck). Samples were stored in a monitored refrigerator at a nominal temperature of 8°C until analysis. Blank samples were prepared by adding concentrated nitric acid to tubes without sample and subsequently diluted in the same manner described above. Multielement calibration standards (ICP multi-element standard solution IV, Merck, Germany, Phosphorus Standard for ICP, Inorganic Ventures, US) were prepared with different concentrations of inorganic elements in the same manner as in blanks and samples. Deionized water (Direct Q UV, Millipore, approximately 18.0 MΩ) was used for preparation of all solutions.

Samples of reference material (Seronorm™ Trace Elements Serum L-1, cat. 201405) (*n* = 3) were prepared in the same manner as the samples. The wavelengths (nm) were Ca 315.887; P 178.284; Na 589.592; K 766.490; Fe 259.94; Mg 280.270; Cu 224.700; Zn 206.200; Sr 421.552. The results of analysis of reference material are presented in Table 11.

**Table 11:** Analysis of reference material Seronorm™ Trace Elements Serum L-1, cat. 201405 using ICP-OES.

**Bioelement**	**Seronorm™ Trace Elements** **Serum L-1 Certified [mg/l]**	**Seronorm™ Trace Elements** **Serum L-1 Measured [mg/l]**	**Recovery (%)**
Ca 315.887	96.00	94.98	98.94
P 178.284	65.00	69.41	106.78
Na 589.592	2998.00	3105.12	103.57
K 766.490	126.00	130.09	103.25
Fe 239.562	1.43	1.38	96.50
Mg 285.213	20.60	22.49	109.17
Cu 224.700	1.88	1.67	89.89

#### Determination of IL-1α, IL-1β, IL-6, TNFα, and IFNγ levels

Blood for determination of selected serum inflammatory markers was collected into serum separation tubes. Serum IL-1α, IL-1β, IL-6, TNFα, and IFNγ levels were measured by immunoassay using ELISA kits (DRG, Germany). The sensitivity of the IL-1α assay was 1.1 pg/ml, the intra- and interassay CV values were <5.4% and <10%, respectively. The sensitivity of the IL-6 assay was 2 pg/ml, the intra- and interassay CV values were 4.2% and 4.4%, respectively. The sensitivity of IL-1β assay was 0.35 pg/ml, the intra- and interassay CV values were 2.3% and 4.9%, respectively. The sensitivity of IFNγ assay was 0.03 IU/ml, the intra- and interassay CV values were 3.2% and 5.8%, respectively. The sensitivity of TNFα assay was 0.7 pg/ml.

#### Determination of CRP levels

Venous blood was collected from each of the volunteers after overnight fasting between 7.00 a.m. and 9.30 a.m. in the morning after a 10 min rest in a sitting position from the antecubital vein using Vacutainer tubes free of Pb (Sarstedt, Germany). The blood was collected in accordance with the relevant rules and procedures concerning collecting, storing, and transporting biological material. The levels of CRP were determined [51].

### Statistical analysis

All quantitative variables were initially assessed using descriptive statistics. Measures of central tendency (mean and median) and variability (standard deviation and quartile deviation), as well as the range of measurement (minimum and maximum) were determined.

Pearson’s r correlation coefficient was used to determine the mutual (non-directional) relationships between the levels of specific proinflammatory cytokines and CRP and the levels of the tested bioelements. The two-tailed null hypothesis of no linear relationship between variables was tested. The same relationship was also studied in the subgroups based on BMI (normal/overweight/obese).

Linear regression was used to estimate the directional effect of specific bioelement levels (independent variable) on proinflammatory cytokine and CRP levels (dependent variable). Standardized regression coefficients (β) were determined for each bioelement to establish the direction and strength of the relationship in relation to individual proinflammatory cytokines and CRP.

All statistical calculations were performed using the statistical package STATISTICA, version 13.3 (TIBCO Software Inc, Palo Alto, California, United States). For all analyses, a two-tailed *P*−value of <0.05 was considered statistically significant.
